# Association between sedentary behaviour, sleep duration, physical activity and mental health among teachers in China during COVID-19: a cross- sectional study

**DOI:** 10.3389/fpubh.2026.1768160

**Published:** 2026-02-25

**Authors:** Yexin Ma, Yuntao Xu, Liyou Wu, Jiabao Guo, Binglin Chen

**Affiliations:** The Second Clinical Medical College, Xuzhou Medical University, Xuzhou, China

**Keywords:** COVID-19, mental health, physical activity, sedentary behaviour, sleep duration, teachers

## Abstract

**Background:**

The COVID-2019 pandemic has led to changes in lifestyles, including sedentary behaviour, sleep duration and physical activity, which have further implications for people’s mental health. However, teachers are an important group whose mental health is often overlooked.

**Objective:**

We aimed to investigate the relationship between sedentary behaviour, sleep duration and physical activity on teachers’ mental health during the COVID-19 pandemic in China.

**Methods:**

This is a cross-sectional study targeting teachers from 10 schools in Sichuan Province with data sourced from the National Population Health Data Center. We used one-way analyses and multifactorial logistic regression and with model adjustment to explore the factors associated with teachers’ mental health. Subgroup analyses were used to further explore the magnitude of the effects of sedentary time, sleep duration and physical activity on teachers’ mental health.

**Results:**

The data suggest that reduced sedentary time (*p* = 0.016; *p* = 0.000) and adequate sleep (*p* = 0.040; p = 0.000) are related to anxiety and depression, whereas frequency of physical activity (*p* = 0.002) may be related to depression. Further analyses found that high frequency of physical activity in the context of long sedentary time and short sleep duration may be associated with anxiety and depression.

**Conclusion:**

Integrated behavioural interventions (sedentary behaviour, sleep duration and physical activity) are potential targets for the prevention and intervention of negative emotions in teachers. However, there is a lack of 24-h behavioural movement guidelines that are more specific to particular groups such as teachers, which is a gap that needs to be addressed in the future.

## 1 Introduction

The global public health landscape has been profoundly reshaped by the COVID-19 pandemic. In the face of thousands of infections, the Chinese government has implemented stringent measures to curtail the virus’s spread. These include inter-city transport restrictions, mandatory home quarantines, centralized isolation of affected individuals, and strict enforcement of social distancing protocols (1), as a consequence, this has triggered a significant transformation in people’s lifestyles. One of the pivotal changes is the 24-h movement behaviour. Extensive research has revealed that it has deteriorated notably during the pandemic (2, 3). People usually experienced longer periods of sedentary time, lower frequency of physical activity and shorter periods of sleep (4, 5). These changes may be related to mental health problems, including anxiety and depression (3, 6–9). One manifestation of these changes is the shift in teaching models from traditional face-to-face instruction to online learning, which may lead to increased periods of prolonged sitting in front of screens for both pupils and teachers, alongside a reduction in physical activity (10, 11).

In China, due to high expectations from society and parents, teachers are facing increasing pressure, the implementation of the “double reduction” policy has further increased the rate of job burnout among teachers, leading to a pessimistic outlook on their mental health (12, 13). Moreover, the dread of COVID-19, coupled with shifts in teaching methodologies, the constant worry of infecting themselves or their students, inadequate sleep, and a lack of physical activity all these factors may collectively contribute to the rising anxiety and depression among teachers (14, 15). Previous researches suggest that physical activity, sleep duration and sedentary behaviour may be effective moderators of mental health (16–19), however the impact of the epidemic has led to disruptions in 24-h patterns of behavioural activity, which may have led to negative impacts on teachers’ mental health. In the cross-sectional studies of teachers at the beginning of the epidemic in China, it was shown that teachers were approximately three times more likely to suffer from anxiety during the epidemic than they were before (20), and more than half of teachers suffer from depression. Chronic mental health problems can lead to physical health problems in teachers. These include cardiovascular disease and metabolic syndrome (21, 22). Moreover, a higher level of depression among teachers can lead to a lower level of well-being among their pupils (23). Therefore, it becomes necessary to study the mental health status of teachers in the specific context of the epidemic.

Despite the fact that up to now a number of existing studies have demonstrated the effects of the triad of sedentary time, physical activity, and sleep duration on mental health (8, 24–26), professional groups represented by teachers are often neglected. Although some studies in recent years have recognized the importance of the teacher population as a special group, for example looking at the impact of physical activity and screen time on aspects of teachers’ mental health (27), they have not considered all three 24-h behavioural factors (28, 29). Consequently, the aim of our study was to investigate whether sedentary behaviour, sleep duration and physical activity are related to teachers’ mental health during the COVID-19 pandemic and aims to provide a reference framework for enhancing the mental wellbeing of the teaching profession.

## 2 Methods

### 2.1 Study design and participants

We obtained data from National Population Health Data Centre-The Science Database of People Mental Health. This database encompasses data from multiple populations, and we have selected the data from the teacher population in 2021 for a cross-sectional study. It primarily comprises 10 schools, which were recruited and included in the study after signing informed consent in Sichuan Province, China. It is a publicly available data, compliance with national regulations on the management of scientific data, open sharing of all data and the signing of agreements on the integrity and confidentiality of data with data management organizations. The study was approved by the Ethics Committee of Xuzhou Medical University (XZHMU-25S3001).

### 2.2 Mental health

The levels of anxiety and depression are measured using Self-rating Anxiety Scale (SAS) and Self-rating depression scale (SDS). SAS is a clinical questionnaire created by Zung (30) to assess anxiety symptoms, it consists of 20 questions, each question has four ratings from 1(none or very few) to 4(most or all). Questions 5, 9, 13, 17, and 19 require reverse scoring. The scores for all questions were added together to obtain the raw score, and the resulting raw score was multiplied by 1.25 and round to the nearest whole number to obtain the criterion score. According to previous study (31), a critical score of 40 for raw scores is the most appropriate for use in research. In our study, therefore, when the criterion score exceeded 50, it signified the presence of anxiety symptoms. SDS is also a scale created by Zung (32) to assess a patient’s depression in the past week, similar to the SAS, it is made up of 20 questions scored from 1 to 4, 10 of the them require reverse scoring and the higher scores indicate more severe depressive symptoms. According to the Chinese norm, when the standardized score is greater than 53, it can indicate depressive symptoms (33, 34). In our study, we have classified anxiety and depression into normal and abnormal categories based on the two cut-off values detailed above.

### 2.3 Primary variables and covariables

Continuous variables were described as mean and standard deviation (SD), including age, Body Mass Index (BMI), sleep duration, weekly class time and teaching age. Categorical variables were described as frequency and ratio, including gender (male and female), education level (vocational high school, vocational college student, undergraduate, graduate student), family economic status (good, average and poor), marital status (married, unmarried and others), self-reported health status (good, average and poor), sleep quality (good, average and poor), smoke (yes or no), drinking (never, 1–2 times/week, 3–4 times/week and 5–6 times/week), physical activity (never, 1–2 times/week, 3–4 times/week and 5–6 times/week), sedentary time (0–2 h, 3–4 h, 5–6 h, and >6 h), work stress (high, average and low), work satisfaction (satisfied, general and unsatisfied), working hours (long, average and short), overtime frequency (more, average and less), age group (primary school, middle school and high school), employment form (tenured teacher, contract teacher, long-term employed teacher, short-term employed teacher and part-time teacher) and professional position (none, master teacher, senior teacher, intermediate teacher, junior senior teacher and junior teacher). To make our study more convenient, we also divided sleep duration into two subgroups (≤7 h and >7 h).

### 2.4 Statistical analyses

We performed descriptive statistics with one-way logistic regression analysis of all variables by using SPSS version 26. To further validate the effects of sedentary time, physical activity, and sleep duration on anxiety and depression, we performed multi-factor logistic regression analysis and made model adjustments using R 4.2.2 software. Model 1 was adjusted for demographic factors, including gender, age, education level, family economic status and marital status. Model 2 was adjusted for health-related factors, including BMI, self-reported health status, sleep duration and sleep quality. Model 3 was adjusted for lifestyle-related factors, including drinking, physical activity and sedentary time. Model 4 was adjusted for work-related factors, including work stress, work satisfaction, working hours, weekly class time, overtime frequency. In addition to this, subgroup analyses based on sedentary time and sleep duration were used to further explore the relationship between the two and physical activity and their effects on anxiety and depression. To assess multicollinearity, we calculated generalized variance-inflation factor (GVIF). If the GVIF (1/2df) < 2, this indicates the presence of multicollinearity in the model. Odds ratios (ORs) and 95% confidential intervals were used to indicate the strength of the relationship. Two-tailed *p*-values <0.05 were considered statistically significant.

## 3 Results

A total of 1,670 teachers were included in this study, as detailed in Table 1. The majority of participants were female (59.6%), with an average age of 37.0 ± 10.2 years. 15.3% had depressive symptoms and 80.7% suffered from anxiety symptoms. A significant portion held a bachelor’s degree (61.0%), were married (76.8%), and reported an average family economic status (81.6%). The average BMI among the teachers was 23.5 kg/m^2^. Over half (51.5%) of the teachers reported their health status as good, while 45.7% reported average sleep quality. In terms of lifestyle-related factors 60.1% did not consume alcohol. Most participants engaged in physical activity 1–2 times a week (54.4%), and a substantial number reported sitting for 0–2 h daily (39.3%). With regards to work-related factors, the majority experienced high levels of work stress (63.8%), yet 50.5% expressed satisfaction with their jobs. The teachers worked longer hours (72.2%) and had less overtime (37.6%). The average weekly class time was 18.7 h.

**Table 1:** Characteristics of study participants.

Variable	*N* (%)or mean±SD	Depression (SDS)		*p*-value	Anxiety (SAS)		*p*-value
		Normal (*n* = 1,415,84.7%)	Abnormal (*n* = 255,15.3%)		Normal (*n* = 322,19.3%)	Abnormal (*n* = 1,348,80.7%)	
Basic information							
Age	37.0 (10.2)			**0.004**			**0.045**
Gender				0.216			0.540
Male	675 (40.4)	563 (83.4)	112 (16.6)		135 (20.0)	540 (80.0)	
Female	995 (59.6)	852 (85.6)	143 (14.4)		187 (18.8)	808 (81.2)	
Education level				0.272			0.217
Vocational High School	68 (4.1)	55 (80.9)	13 (19.1)		22 (32.4)	46 (67.6)	
Vocational College Student	576 (34.5)	482 (83.7)	94 (16.3)		103 (17.9)	473 (82.1)	
Undergraduate	1,018 (61.0)	872 (85.7)	146 (14.3)		196 (19.3)	822 (80.7)	
Graduate Student	8 (0.5)	6 (75.0)	2 (25.0)		1 (12.5)	7 (87.5)	
Family economic status				**0.001**			0.524
Good	128 (6.7)	112 (87.5)	16 (12.5)		31 (24.2)	97 (75.8)	
Average	1,362 (81.6)	1,167 (85.7)	195 (14.3)		247 (18.1)	1,115 (81.9)	
Poor	180 (10.8)	136 (75.6)	44 (24.4)		44 (24.4)	136 (75.6)	
Marital status				0.629			**0.046**
Married	1,282 (76.8)	1,088 (84.9)	194 (15.1)		236 (18.4)	1,046 (81.6)	
Unmarried	353 (21.1)	299 (84.7)	54 (15.3)		75 (21.2)	278 (78.8)	
Others	35 (2.1)	28 (80.0)	7 (20.0)		11 (31.4)	24 (68.6)	
Health-related							
BMI	23.5 (5.4)			0.753			0.518
Self-reported health status				**0.000**			0.073
Good	859 (51.5)	800 (93.1)	59 (6.9)		173 (20.1)	686 (79.9)	
Average	682 (40.8)	549 (80.5)	133 (19.5)		133 (19.5)	549 (80.5)	
Poor	129 (7.8)	66 (51.2)	63 (48.8)		16 (12.4)	113 (87.6)	
Sleep duration	7.2 (1.0)			**0.000**			**0.040**
Sleep quality				**0.000**			**0.022**
Good	574 (34.4)	537 (93.6)	37 (6.4)		109 (19.0)	465 (81.0)	
Average	764 (45.7)	663 (86.8)	101 (13.2)		174 (22.8)	590 (77.2)	
Poor	332 (19.9)	215 (64.8)	117 (35.2)		39 (11.7)	293 (88.3)	
Lifestyle-related							
Drinking				0.133			0.687
Never	1,003 (60.1)	860 (85.7)	143 (14.3)		196 (19.5)	807 (80.5)	
1–2 times/week	589 (35.3)	490 (83.2)	99 (16.8)		106 (18.0)	483 (82.0)	
3–4 times/week	51 (3.1)	45 (88.2)	6 (11.8)		13 (25.5)	38 (74.5)	
5–6 times/week	27 (1.6)	20 (74.1)	7 (25.9)		7 (25.9)	20 (74.1)	
Physical activity				**0.002**			0.082
Never	414 (24.8)	326 (78.7)	88 (21.3)		90 (21.7)	324 (78.3)	
1–2 times/week	909 (54.4)	786 (86.5)	123 (13.5)		174 (19.1)	735 (80.9)	
3–4 times/week	216 (12.9)	187 (86.6)	29 (13.4)		37 (17.1)	179 (82.9)	
5–6 times/week	131 (7.8)	116 (88.5)	15 (11.5)		21 (16.0)	110 (84.0)	
Sedentary time				**0.000**			**0.016**
0 to 2 h	656 (39.3)	590 (89.9)	66 (10.1)		144 (22.0)	512 (78.0)	
3 to 4 h	515 (30.8)	446 (86.6)	69 (13.4)		91 (17.7)	424 (82.3)	
5 to 6 h	319 (19.1)	259 (81.2)	60 (18.8)		62 (19.4)	257 (80.6)	
>6 h	180 (10.8)	120 (66.7)	60 (33.3)		25 (13.9)	155 (86.1)	
Work-related							
Work stress				**0.000**			0.096
High	1,066 (63.8)	845 (79.3)	221 (20.7)		189 (17.7)	877 (82.2)	
Average	553 (33.1)	521 (94.2)	32 (5.8)		121 (21.9)	432 (78.1)	
Low	51 (3.0)	49 (96.0)	2 (4.0)		12 (23.5)	39 (76.5)	
Work satisfaction				**0.000**			0.975
Satisfied	844 (50.5)	763 (90.4)	81 (9.6)		155 (18.4)	689 (81.6)	
General	666 (39.9)	557 (83.6)	109 (16.4)		135 (20.3)	531 (79.7)	
Unsatisfied	160 (9.5)	95 (59.4)	65 (40.6)		32 (20.0)	128 (80.0)	
Working hours				**0.000**			**0.000**
Long	1,206 (72.2)	991 (82.2)	215 (17.8)		205 (17.0)	1,001 (83.0)	
Average	346 (20.7)	314 (90.8)	32 (9.2)		87 (25.1)	259 (74.9)	
Short	118 (7.0)	110 (93.2)	8 (6.8)		30 (25.4)	88 (74.6)	
Overtime frequency				**0.000**			0.161
More	481 (28.8)	373 (77.5)	108 (22.5)		85 (17.7)	396 (82.3)	
Average	561 (33.6)	481 (85.7)	80 (14.3)		110 (19.6)	451 (80.4)	
less	628 (37.6)	561 (89.3)	67 (10.7)		127 (20.2)	501 (79.8)	
Weekly class time	18.7 (10.7)			0.051			0.431

BMI, body mass index. Bold values indicate *p* < 0.05.

As shown in Table 2, GVIF (1/2df) < 2 for all cases, which rules out collinearity among the factors in the model. In the one-way logistic regression analysis, we have found that the correlates of anxiety and depression are age, family economic status, etc. (more details in Table 1) Following adjustments to the models, as presented in Table 3, we observed that sitting for 3 to 4 h did not significantly influence anxiety or depression. However, a sitting duration of 5–6 h is statistically significant for depression. Physical activity is associated with a lower prevalence of depression, which is only significant in Model 1 and Model 3. When sleep duration exceeds 7 hours, the probability of both anxiety and depression levels are significantly reduced (*p* < 0.05).

**Table 2:** GVIF (1/2df) values between different model factors.

Variable name/GVIF(1/2df)	Anxiety	Depression	Anxiety	Depression	Anxiety	Depression
	Sedentary time		Physical activity		Sleep duration	
Model 1						
	1.014	1.014	1.021	1.023	1.030	1.022
Gender	1.118	1.134	1.141	1.149	1.120	1.132
Age	1.419	1.483	1.411	1.491	1.425	1.494
Education level	1.037	1.045	1.034	1.041	1.033	1.039
Marital status	1.115	1.131	1.114	1.132	1.113	1.134
Family economic status	1.012	1.012	1.013	1.013	1.012	1.011
Model 2						
	1.009	1.007	1.016	1.012	1.079	1.077
BMI	1.003	1.003	1.006	1.006	1.003	1.002
Self-reported health status	1.060	1.069	1.062	1.070	1.057	1.065
Sleep duration	1.082	1.080	1.079	1.077	NA	NA
Sleep quality	1.063	1.074	1.064	1.074	1.062	1.072
Model 3						
	1.003	1.003	1.009	1.009	1.016	1.008
Drinking	1.008	1.009	1.008	1.009	1.010	1.012
Sedentary time	NA	NA	1.003	1.003	1.006	1.004
Physical activity	1.009	1.009	NA	NA	1.008	1.011
Model 4						
	1.018	1.014	1.016	1.017	1.057	1.034
Work stress	1.063	1.050	1.062	1.051	1.063	1.051
Work satisfaction	1.032	1.026	1.036	1.030	1.035	1.026
Working hours	1.072	1.064	1.070	1.062	1.071	1.064
Weekly class time	1.027	1.027	1.024	1.024	1.023	1.025
Overtime frequency	1.054	1.046	1.053	1.044	1.059	1.044

Sleep duration ≤7				
	Anxiety	Depression	Anxiety	Depression
	Sedentary time		Physical activity	
Model 1				
	1.015	1.015	1.025	1.028
Gender	1.148	1.151	1.163	1.165
Age	1.523	1.534	1.545	1.577
Education level	1.045	1.058	1.041	1.046
Marital status	1.144	1.144	1.144	1.146
Family economic status	1.015	1.013	1.014	1.012
Model 2				
	1.008	1.006	1.012	1.010
BMI	1.002	1.004	1.005	1.005
Self-reported health status	1.036	1.038	1.036	1.038
Sleep quality	1.034	1.035	1.034	1.036
Model 3				
	1.005	1.004	1.015	1.014
Drinking	1.015	1.013	1.015	1.013
Sedentary time	NA	NA	1.005	1.004
Physical activity	1.015	1.014	NA	NA
Model 4				
	1.023	1.019	1.017	1.020
Work stress	1.056	1.041	1.054	1.042
Work satisfaction	1.029	1.024	1.031	1.028
Working hours	1.077	1.066	1.075	1.064
Weekly class time	1.038	1.036	1.039	1.036
Overtime frequency	1.064	1.050	1.060	1.051

Sleep duration >7				
	Anxiety	Depression	Anxiety	Depression
	Sedentary time		Physical activity	
Model 1				
	1.017	1.018	1.030	1.035
Gender	1.107	1.117	1.133	1.144
Age	1.364	1.441	1.360	1.442
Education level	1.042	1.049	1.043	1.053
Marital status	1.096	1.125	1.101	1.131
Family economic status	1.016	1.020	1.020	1.025
Model 2				
	1.007	1.010	1.021	1.027
BMI	1.013	1.011	1.012	1.021
Self-reported health status	1.101	1.126	1.110	1.129
Sleep quality	1.099	1.126	1.105	1.130
Model 3				
	1.004	1.004	1.005	1.008
Drinking	1.005	1.006	1.005	1.006
Sedentary time	NA	NA	1.004	1.004
Physical activity	1.005	1.008	NA	NA
Model 4				
	1.018	1.031	1.023	1.031
Work stress	1.059	1.067	1.060	1.067
Work satisfaction	1.030	1.042	1.035	1.048
Working hours	1.066	1.095	1.067	1.093
Weekly class time	1.042	1.114	1.031	1.111
Overtime frequency	1.046	1.061	1.048	1.056

Sedentary time ≤4				
	Anxiety	Depression	Anxiety	Depression
	Sleep duration		Physical activity	
Model 1				
	1.028	1.025	1.026	1.025
Gender	1.108	1.121	1.136	1.143
Age	1.381	1.485	1.368	1.475
Education level	1.029	1.039	1.031	1.040
Marital status	1.106	1.133	1.108	1.130
Family economic status	1.012	1.013	1.015	1.014
Model 2				
	1.081	1.076	1.017	1.015
BMI	1.006	1.008	1.010	1.014
Self-reported health status	1.051	1.049	1.057	1.056
Sleep duration	NA	NA	1.082	1.078
Sleep quality	1.057	1.058	1.059	1.060
Model 3				
	1.009	1.004	1.007	1.007
Drinking	1.007	1.009	1.007	1.007
Physical activity	1.008	1.010	NA	NA
Model 4				
	1.052	1.033	1.017	1.021
Work stress	1.060	1.044	1.060	1.045
Work satisfaction	1.034	1.030	1.036	1.032
Working hours	1.072	1.061	1.072	1.059
Weekly class time	1.028	1.031	1.031	1.030
Overtime frequency	1.046	1.040	1.048	1.042

Sedentary time >4				
	Anxiety	Depression	Anxiety	Depression
	Sleep duration		Physical activity	
Model 1				
	1.028	1.026	1.021	1.310
Gender	1.168	1.166	1.177	1.186
Age	1.557	1.567	1.562	1.594
Education level	1.044	1.057	1.044	1.062
Marital status	1.147	1.152	1.146	1.155
Family economic status	1.015	1.016	1.014	1.014
Model 2				
	1.050	1.068	1.017	1.022
BMI	1.006	1.010	1.048	1.066
Self-reported health status	1.059	1.103	1.007	1.012
Sleep duration	NA	NA	1.064	1.108
Sleep quality	1.060	1.105	1.067	1.107
Model 3				
	1.004	1.002	1.010	1.010
Drinking	1.011	1.011	1.010	1.010
Physical activity	1.011	1.012	NA	NA
Model 4				
	1.052	1.055	1.026	1.033
Work stress	1.051	1.070	1.051	1.069
Work satisfaction	1.045	1.057	1.050	1.064
Working hours	1.072	1.075	1.074	1.078
Weekly class time	1.060	1.060	1.062	1.066
Overtime frequency	1.060	1.060	1.062	1.062

**Table 3:** Effects of sedentary time, physical activity and sleep duration on teachers’ anxiety and depression.

Subgroup	Model 1 OR, 95% CI		Model 2 OR, 95% CI		Model 3 OR, 95% CI		Model 4 OR, 95% CI	
	Anxiety	Depression	Anxiety	Depression	Anxiety	Depression	Anxiety	Depression
Sedentary time								
0 to 2 h	Ref	Ref	Ref	Ref	Ref	Ref	Ref	Ref
3 to 4 h	1.25 (0.93,1.69)	1.33 (0.92, 1.93)	1.30 (0.97, 1.75)	1.23 (0.84, 1.80)	1.32 (0.99, 1.78)	1.38 (0.96, 1.99)	1.24 (0.92, 1.68)	1.27 (0.87, 1.86)
*p*-value	0.147	0.125	0.085	0.297	0.064	0.078	0.154	0.220
5 to 6 h	1.12 (0.79, 1.58)	1.91 (1.29, 2.82)	1.11 (0.79, 1.56)	1.57 (1.04, 2.36)	1.18 (0.85, 1.66)	1.97 (1.34, 2.89)	1.12 (0.80, 1.59)	1.67 (1.11, 2.51)
*p*-value	0.531	**0.001**	0.558	**0.031**	0.326	**<0.001**	0.510	**0.014**
>6 h	1.73 (1.10, 2.82)	4.27 (2.83, 6.46)	1.54 (0.97, 2.51)	2.98 (1.91, 4.64)	1.80 (1.15, 2.91)	4.19 (2.80, 6.29)	1.59 (1.00, 2.61)	2.79 (1.79, 4.34)
*p*-value	**0.023**	**<0.001**	0.075	**<0.001**	**0.013**	**<0.001**	0.584	**<0.001**
Physical activity								
Never	Ref	Ref	Ref	Ref	Ref	Ref	Ref	Ref
1–2 times/week	1.16 (0.86, 1.55)	0.54 (0.40, 0.74)	1.27 (0.94, 1.70)	0.78 (0.55, 1.07)	1.19 (0.89, 1.59)	0.60 (0.44, 0.83)	1.23 (0.91, 1.66)	0.80 (0.57, 1.13)
*p*-value	0.323	**<0.001**	0.111	0.119	0.234	**0.002**	0.167	0.199
3–4 times/week	1.39 (0.90, 2.19)	0.48 (0.29, 0.77)	1.48 (0.96, 2.31)	0.85 (0.51, 1.38)	1.40 (0.91, 2.17)	0.59 (0.36, 0.93)	1.46 (0.95, 2.28)	0.80 (0.48, 1.30)
*p*-value	0.146	**0.003**	0.079	0.523	0.128	**0.027**	0.091	0.373
5–6 times/week	1.57 (0.92, 2.76)	0.38 (0.20, 0.68)	1.65 (0.98, 2.90)	0.79 (0.40, 1.47)	1.51 (0.90, 2.62)	0.49 (0.26, 0.87)	1.52 (0.90, 2.66)	0.75 (0.39, 1.39)
*p*-value	0.105	**0.002**	0.069	0.469	0.126	**0.020**	0.126	0.376
Sleep duration								
≤7 h	Ref	Ref	Ref	Ref	Ref	Ref	Ref	Ref
>7 h	0.76 (0.59, 0.98)	0.40 (0.29, 0.54)	0.76 (0.58, 1.00)	0.67 (0.47, 0.94)	0.72 (0.56, 0.92)	0.43 (0.31, 0.59)	0.76 (0.58, 0.98)	0.55 (0.39, 0.77)
*p*-value	**0.033**	**<0.001**	**0.044**	**0.024**	**0.009**	**<0.001**	**0.036**	**<0.001**

Model 1 was adjusted for demographics, including gender, age, education level, family economic status and marital status.

Model 2 was adjusted for health-related factors, including BMI, self-reported health status, sleep duration and sleep quality.

Model 3 was adjusted for lifestyle factors, including drinking, physical activity and sedentary time.

Model 4 was adjusted for work-related factors, including work stress, work satisfaction, working hours, weekly class time, overtime frequency.

BMI, body mass index; OR, odds ratio; CI, confidence interval. Bold values indicate *p* < 0.05.

Table 4 shows that the prevalence of depression almost doubles when sitting for more than 6 h compared to 5 to 6 h. When the frequency of physical activity was 5–6 times/week, the probability of experiencing anxiety increases by nearly twofold compared to when 1–2 times/week. Furthermore, when sleep duration >7 h and sedentary time >6 h, the prevalence of anxiety and depression rises significantly.

**Table 4:** Correlations between sedentary behaviour, sleep duration and physical activity when grouped by sleep duration.

Subgroup	Model 1 OR, 95% CI		Model 2 OR, 95% CI		Model 3 OR, 95% CI		Model 4 OR, 95% CI	
	Anxiety	Depression	Anxiety	Depression	Anxiety	Depression	Anxiety	Depression
Sleep duration ≤7								
Sedentary time								
0 to 2 h	Ref	Ref	Ref	Ref	Ref	Ref	Ref	Ref
3 to 4 h	1.11 (0.72, 1.72)	1.42 (0.92, 2.22)	1.11 (0.72, 1.70)	1.29 (0.82, 2.04)	1.14 (0.75, 1.76)	1.45 (0.94, 2.24)	1.13 (0.74, 1.76)	1.36 (0.86, 2.15)
*p*-value	0.630	0.116	0.641	0.268	0.540	0.094	0.570	0.185
5 to 6 h	0.83 (0.52, 1.31)	1.83 (1.15, 2.92)	0.75 (0.48, 1.19)	1.52 (0.94, 2.47)	0.81 (0.52, 1.26)	1.89 (1.20, 2.98)	0.80 (0.51, 1.27)	1.63 (1.01, 2.64)
*p*-value	0.410	**0.011**	0.218	0.087	0.343	**0.006**	0.343	**0.045**
>6 h	1.11 (0.64, 1.99)	3.94 (2.42, 6.45)	0.97 (0.56, 1.72)	2.90 (1.75, 4.84)	1.13 (0.66, 1.99)	3.82 (2.38, 6.16)	1.08 (0.61, 1.95)	2.57 (1.55, 4.28)
*p*-value	0.706	**<0.001**	0.917	**<0.001**	0.665	**<0.001**	0.789	**<0.001**
Physical activity								
Never	Ref	Ref	Ref	Ref	Ref	Ref	Ref	Ref
1–2 times/week	1.15 (0.78, 1.71)	0.64 (0.44, 0.92)	1.23 (0.84, 1.81)	0.80 (0.55, 1.17)	1.16 (0.79, 1.71)	0.71 (0.49, 1.03)	1.17 (0.78, 1.74)	0.87 (0.59, 1.30)
*p*-value	0.461	**0.016**	0.287	0.254	0.447	0.073	0.436	0.500
3–4 times/week	1.17 (0.65, 2.16)	0.71 (0.40, 1.23)	1.21 (0.69, 2.20)	1.00 (0.57, 1.75)	1.20 (0.68, 2.20)	0.89 (0.51, 1.52)	1.18 (0.67, 2.17)	1.09 (0.61, 1.92)
*p*-value	0.617	0.233	0.511	0.982	0.530	0.668	0.576	0.759
5–6 times/week	3.17 (1.27, 9.67)	0.48 (0.21, 0.99)	3.11 (1.26, 9.43)	0.78 (0.33, 1.69)	3.06 (1.25, 9.23)	0.57 (0.26, 1.17)	2.73 (1.11, 8.27)	0.87 (0.38, 1.84)
*p*-value	**0.023**	0.057	**0.024**	0.540	**0.025**	0.146	**0.045**	0.724
Sleep duration >7								
Sedentary time								
0 to 2 h	Ref	Ref	Ref	Ref	Ref	Ref	Ref	Ref
3 to 4 h	1.36 (0.89, 2.10)	0.99 (0.48, 2.00)	1.47 (0.97, 2.23)	1.09 (0.52, 2.28)	1.43 (0.95, 2.18)	1.02 (0.50, 2.02)	1.34 (0.88, 2.07)	0.91 (0.42, 1.94)
*p*-value	0.153	0.974	0.072	0.814	0.090	0.965	0.178	0.813
5 to 6 h	1.63 (0.95, 2.90)	1.76 (0.81, 3.71)	1.83 (1.05, 3.18)	1.72 (0.79, 3.76)	1.81 (1.06, 3.19)	1.70 (0.77, 3.39)	1.69 (0.98, 3.04)	1.75 (0.75, 4.00)
*p*-value	0.087	0.145	**0.033**	0.175	**0.034**	0.190	0.067	0.187
>6 h	4.09 (1.57, 14.05)	3.67 (1.52, 8.43)	4.03 (1.39, 11.65)	3.61 (1.43, 9.08)	3.99 (1.55, 13.58)	3.56 (1.56, 8.63)	3.86 (1.46, 13.35)	3.48 (1.31, 8.90)
*p*-value	**0.010**	**0.003**	**0.010**	**0.006**	**0.010**	**0.002**	**0.014**	**0.010**
Physical activity								
Never	Ref	Ref	Ref	Ref	Ref	Ref	Ref	Ref
1–2 times/week	1.29 (0.81, 2.03)	0.43 (0.23, 0.82)	1.35 (0.85, 2.14)	0.74 (0.37, 1.48)	1.35 (0.85, 2.11)	0.46 (0.25, 0.87)	1.38 (0.86, 2.19)	0.68 (0.34, 1.37)
*p*-value	0.278	**0.009**	0.207	0.401	0.200	**0.015**	0.179	0.265
3–4 times/week	1.97 (1.01, 3.95)	0.23 (0.07, 0.61)	1.83 (0.94, 3.58)	0.49 (0.16, 1.46)	1.82 (0.96, 3.57)	0.26 (0.08, 0.69)	1.96 (1.01, 3.92)	0.34 (0.10, 1.02)
*p*-value	0.050	**0.006**	0.075	0.198	0.072	**0.012**	0.050	0.068
5–6 times/week	1.24 (0.61, 2.60)	0.35 (0.10, 0.98)	1.17 (0.57, 2.36)	0.76 (0.25, 2.32)	1.19 (0.61, 2.42)	0.48 (0.15, 1.28)	1.09 (0.54, 2.25)	0.54 (0.15, 1.63)
*p*-value	0.558	0.060	0.672	0.626	0.615	0.169	0.822	0.300

Model 1 was adjusted for demographics, including gender, age, education level, family economic status and marital status.

Model 2 was adjusted for health-related factors, including BMI, self-reported health status, sleep duration and sleep quality.

Model 3 was adjusted for lifestyle factors, including drinking, physical activity and sedentary time.

Model 4 was adjusted for work-related factors, including work stress, work satisfaction, working hours, weekly class time, overtime frequency.

BMI, body mass index; OR, odds ratio; CI, confidence interval. Bold values indicate *p* < 0.05.

Based on our prior findings, we found that 4 h of sitting was the node where the results were significant or not, so we used 4 h as the basis for grouping. As Table 5 illustrates, when the sedentary time was ≤4 h, sleeping for more than 7 h is associated with a lower probability of anxiety and depression. Conversely, when the sedentary time >4 h, sleeping >7 h notably decreased the likelihood of depression in only Model 1 (OR = 0.43, CI = 0.25–0.72) and Model 3 (OR = 0.43, CI = 0.26–0.71).

**Table 5:** Correlations between sedentary behaviour, sleep duration and physical activity when grouped by sedentary time.

Subgroup	Model 1 OR, 95% CI		Model 2 OR, 95% CI		Model 3 OR, 95% CI		Model 4 OR, 95% CI	
	Anxiety	Depression	Anxiety	Depression	Anxiety	Depression	Anxiety	Depression
Sedentary time ≤4								
Sleep duration								
≤7 h	Ref	Ref	Ref	Ref	Ref	Ref	Ref	Ref
>7 h	0.66 (0.49, 0.89)	0.42 (0.28, 0.63)	0.61 (0.45, 0.84)	0.74 (0.47, 1.15)	0.58 (0.43, 0.77)	0.41 (0.27, 0.61)	0.64 (0.47, 0.87)	0.54 (0.35, 0.83)
*p*-value	**0.007**	**<0.001**	**0.002**	**<0.001**	**<0.001**	**<0.001**	**0.005**	**0.005**
Physical activity								
Never	Ref	Ref	Ref	Ref	Ref	Ref	Ref	Ref
1–2 times/week	1.15 (0.80, 1.65)	0.72 (0.46, 1.12)	1.29 (0.90, 1.83)	1.00 (0.64, 1.61)	1.20 (0.84, 1.70)	0.73 (0.48, 1.12)	1.33 (0.92, 1.91)	1.08 (0.68, 1.75)
*p*-value	0.436	0.137	0.167	0.988	0.305	0.143	0.130	0.746
3–4 times/week	1.18 (0.70, 2.03)	0.67 (0.34, 1.27)	1.25 (0.76, 2.11)	1.12 (0.56, 2.17)	1.17 (0.72, 1.96)	0.73 (0.38, 1.34)	1.31 (0.79, 2.21)	1.07 (0.54, 2.09)
*p*-value	0.530	0.228	0.385	0.736	0.517	0.322	0.307	0.823
5–6 times/week	1.53 (0.83, 2.95)	0.39 (0.15, 0.90)	1.65 (0.90, 3.15)	0.74 (0.27, 1.76)	1.45 (0.81, 2.71)	0.41 (0.16, 0.91)	1.60 (0.87, 3.03)	0.68 (0.26, 1.58)
*p*-value	0.186	**0.037**	0.115	0.517	0.228	**0.041**	0.141	0.392
Sedentary time >4								
Sleep duration								
≤7 h	Ref	Ref	Ref	Ref	Ref	Ref	Ref	Ref
>7 h	1.32 (0.78, 2.27)	0.43 (0.25, 0.72)	1.43 (0.84, 2.50)	0.65 (0.37, 1.12)	1.26 (0.76, 2.14)	0.43 (0.26, 0.71)	1.30 (0.76, 2.27)	0.57 (0.32, 1.00)
*p*-value	0.309	**0.002**	0.197	0.127	0.384	**0.001**	0.352	0.051
Physical activity								
Never	Ref	Ref	Ref	Ref	Ref	Ref	Ref	Ref
1–2 times/week	1.19 (0.70, 2.01)	0.44 (0.27, 0.71)	1.23 (0.72, 2.09)	0.60 (0.36, 0.98)	1.16 (0.69, 1.96)	0.49 (0.30, 0.78)	1.11 (0.65, 1.89)	0.65 (0.39, 1.10)
*p*-value	0.516	**0.001**	0.433	**0.043**	0.569	**0.003**	0.703	0.106
3–4 times/week	2.42 (1.03, 6.43)	0.39 (0.18, 0.82)	2.22 (0.95, 5.84)	0.55 (0.25, 1.15)	2.19 (0.95, 5.71)	0.44 (0.21, 0.88)	2.09 (0.89, 5.56)	0.62 (0.28, 1.34)
*p*-value	0.056	**0.015**	0.081	0.124	0.083	**0.025**	0.113	0.238
5–6 times/week	2.14 (0.72, 7.91)	0.55 (0.20, 1.34)	1.94 (0.65, 7.27)	1.03 (0.36, 2.78)	1.76 (0.62, 6.35)	0.65 (0.25, 1.53)	1.33 (0.45, 4.92)	1.22 (0.44, 3.20)
*p*-value	0.203	0.204	0.272	0.951	0.330	0.347	0.532	0.690

Model 1 was adjusted for demographics, including gender, age, education level, family economic status and marital status.

Model 2 was adjusted for health-related factors, including BMI, self-reported health status, sleep duration and sleep quality.

Model 3 was adjusted for lifestyle factors, including drinking, physical activity and sedentary time.

Model 4 was adjusted for work-related factors, including work stress, work satisfaction, working hours, weekly class time, overtime frequency.

BMI, body mass index, OR, odds ratio; CI, confidence interval. Bold values indicate *p* < 0.05.

## 4 Discussion

The outbreak of COVID-19 has led to significant changes in people’s learning and lifestyles. Due to the lockdown measures taken by the government, many schools have had to adopt online teaching models (35). Although the present study did not collect specific data on the duration of isolation or the intensity of remote teaching, the outbreak of COVID-19 likely served as a significant contextual factor. During this period, the shift to online teaching models and government lockdown measures presented teachers with unique challenges, such as the need to rapidly learn new techniques and balance work-life boundaries (36–38). These contextual factors may have contributed to the increased work pressure and mental health issues observed in our results, consistent with broader trends reported during the pandemic. Our cross-sectional study delved into exploring the intricate interplay between sedentary behaviour, physical activity, and sleep duration on the psychological well-being of teachers in China during the pandemic context. The study findings underscored the significant associations among these three factors and the prevalent mental health issues of anxiety and depression among teachers.

Our research indicates that prolonged periods of sitting are associated with a higher probability of anxiety and depression, a finding that aligns with prior researches (39–41). The subgroup analyses in Table 4 show that when sleep duration was ≤7 h, more sedentary time was associated with an increased probability of depression, and when sleep duration was >7 h, sedentary time >6 h was associated with an increased probability of both anxiety and depression. In Table 5, when sedentary time is ≤4 h, adequate sleep time is associated with a lower prevalence of anxiety and depression, whereas when sedentary time is >4 h, the results are only significant for depression in some models and lead to a higher probability of anxiety. This suggest that adequate sleep may not be able to reverse the negative effects of sedentary time. Therefore, one of the important measures to improve teachers’ mental health is to reduce their sedentary time.

Sedentary behaviour includes sitting for various purposes, including work and electronic screen-based activities and so on. Because of the inventive and control measures taken by the Government during the pandemic, some schools having to close and thus switch to a home-based online teaching model (14), this has led to a significant increase in the amount of time teachers spend on the screen, which further increases sedentary time. Therefore, in the special context of the pandemic, one way to reduce teachers’ sedentary behaviour is by reducing screen time. Screen-based activities lead to increased arousal in the central nervous system and less social interaction and a withdrawal from relationships, which can lead to an increased risk of anxiety and depression (42–44). In a study of Brazilian teachers, they were more likely to report a very low quality of life and to feel sad and anxious on a regular basis if they used computers/tablets for more than 5 hours a day (27). Relevant departments can set time limits for online classes, meetings, to avoid unlimited screen time and teachers can be encouraged to use monitoring software on their own devices, such as time reminder timers (27), thereby reducing the sedentary time. In addition, a German study on children found that the use of standing desks in the classroom was effective in reducing sedentary time (45), schools and educational institutions can also use standing desks or liftable desks and chairs in the office to help teachers reduce sedentary behaviour.

Sedentary behaviour, physical activity and sleep duration are integral components of the 24-h movement behaviour framework. Given that physical activity in this study was assessed as weekly frequency, these behaviours represent distinct yet interrelated dimensions of lifestyle. An increase in sedentary behaviour often reflects a sedentary lifestyle pattern, which may be associated with lower physical activity frequency or reduced sleep duration (46, 47). In our study, almost 80% of the teachers were not physically active or were only physically active 1–2 times/week. According to the results of univariate analysis, physical activity was significantly associated with depression, and although only in models 1 and 3 was the effect on depression statistically significant after modeling adjustments, we still found a decrease in the probability of depression as the frequency of weekly physical activity increased. Our findings emphasize that teachers should be actively encouraged to be physically active. Physical activities can trigger various biochemical changes in the brain, including the release of endorphins and the transmission of monoamine neurotransmitters, thereby improving mental health (48).

However, we found in Table 4 that physical activity at 5–6 times/week was associated with increased anxiety when sleep duration was ≤7 h, although physical activity at 1–2 times/week and 3–4 times/week was not significantly associated with anxiety, we observed that the likelihood of anxiety increased as the frequency of weekly physical activity increased. Moreover, the anxiety-inducing effect of physical activity at 5–6 times/week was reduced when sleep duration was >7 h. In Table 5, we found that physical activity at 5–6 times/week was not significantly associated with a reduction in anxiety and depression when sedentary time was > 4 h and there is a slight increase in its probability compared to 1–2 times/week. Previous studies have shown that more exercise is not always better (49, 50). These findings may suggest the need to focus on body conditioning and choosing the right frequency when performing physical activities. Therefore, it is imperative that the focus is shifted toward interventions for teachers’ integrated behavioural patterns, as opposed to merely increasing the frequency of physical activity to cope with teachers’ negative emotions. Of course, we also need to consider the uniqueness of the teaching profession and make adjustments based on actual situations.

Our study showed that greater than 7 h of sleep resulted in lower probability of anxiety and depression, same results as a study in Ningbo, China (51), based on two recent Chinese cross-sectional studies, an excessive amount of sleep was also discovered to heighten the probability of experiencing anxiety and depression (52, 53). Therefore, further research is needed to determine how many hours of sleep are critical for the development of teachers’ anxiety and depression.

This study focuses on the overlooked issue of teachers’ mental health during the epidemic. It is the first to examine the impact of three factors of 24-h movement behavioural and the interrelationships between them on teachers’ mental health in China. It also indicates that physical activity must be maintained at a certain frequency to effectively improve mental health. However, several limitations need to be noticed. Firstly, cross-sectional study design has its own limitations. There may be a reverse causality between the results. Secondly, the classification of physical activity intensity relied on a verbal questionnaire, as opposed to the more robust International Physical Activity Questionnaire or other commonly used standardized questionnaires, which may lead to less accurate acquisition of physical activity frequency, thus potentially contributing to fewer statistically significant results regarding the impact of physical activity on mental health. In addition, physical activity is measured through frequency, this may lead to confusion between intensity and frequency. Despite the same frequency of physical activity per week, there may be significant differences in duration. Meanwhile, investigating physical activity, sedentary time, and sleep duration through specific questions may introduce self-reported recall bias in the subjects, affecting the accuracy of the results. Thirdly, the categorization of anxiety and depression levels in this study led to a substantial disparity between the number of individuals classified as normal and those identified as experiencing non-normal levels, thereby introducing a degree of bias into the results. Fourthly, the limitations imposed by the database’s own data volume and the restricted nature of the data collected within it, for example, the units of measurement for physical activity and sedentary time are not unified, which limits the generalisability of the research. Future research may employ longitudinal cohort studies to investigate the causal relationships among these three factors and their impact on teachers’ mental wellbeing, while expanding the study population to encompass a broader geographical scope. Although our study categorised sedentary time, sleep duration and physical activity, the classification criteria and methodology were not rigorous due to database limitations. Future research can adopt more scientific measurements and classifications when studying sedentary behaviour, physical activity, and sleep duration.

## 5 Conclusion

This study examined an association between sedentary behaviour, sleep duration, physical activity and mental health among teachers. The results indicates that extended periods of sedentary time are closely linked to anxiety and depression among teachers, sufficient sleep significantly reduces the likelihood of experiencing these mental health issues and appropriate physical activity may help to reduce the likelihood of depression. However, high frequency of physical activity in the context of sedentary and sleep deprivation is associated with an increase in the incidence of anxiety. Therefore, teachers may need to reduce sedentary behaviour, increase sleep duration, and maintain regular physical activity to ensure their mental health and comprehensive behavioural interventions for the prevention of negative emotions among teachers are important. Our study may be able to provide data to support the development of precise behavioural guidelines for groups of teachers. Given the cross-sectional design of this study, all findings only reveal statistical associations and cannot infer causal relationships and future longitudinal studies are warranted to clarify the directionality of these associations and disentangle potential reverse-causal pathways.

## Data Availability

The datasets presented in this study can be found in online repositories. The names of the repository/repositories and accession number(s) can be found in the article/supplementary material.
